# Craniomaxillofacial-Derived MSCs in Congenital Defect Reconstruction

**DOI:** 10.3390/biom15070953

**Published:** 2025-06-30

**Authors:** Xiaona Song, Linlin Peng, Zhuan Bian, Wei Yin

**Affiliations:** State Key Laboratory of Oral & Maxillofacial Reconstruction and Regeneration, Key Laboratory of Oral Biomedicine Ministry of Education, Hubei Key Laboratory of Stomatology, School & Hospital of Stomatology, Wuhan University, Wuhan 430079, China; 2022203040014@whu.edu.cn (X.S.); 2023183040036@whu.edu.cn (L.P.)

**Keywords:** craniomaxillofacial-derived MSCs, congenital defects, tissue regeneration, immunogenicity

## Abstract

Tissue defects resulting from craniomaxillofacial congenital developmental anomalies significantly compromise both the physical and psychological health of patients. Due to the constraints of autologous and allogeneic transplantation, stem cell-based regenerative therapies present a promising alternative. As a crucial source of cell therapy, mesenchymal stem cells (MSCs) are widely employed for tissue regeneration on account of their exceptional proliferative capacity and multidirectional differentiation potential. Nevertheless, several challenges remain in clinical application, such as the immunogenicity, long-term safety, and therapeutic efficacy. This review centers on the application of craniomaxillofacial MSCs in the treatment of craniomaxillofacial congenital defects and the challenges confronted in regenerative therapy, aiming to provide new perspectives for the clinical management of these conditions.

## 1. Introduction

Craniomaxillofacial development is one of the most complex biological processes, with associated congenital malformations accounting for approximately one-third of all congenital defects, such as cleft lip and palate and craniosynostosis [1]. The majority of the oral and craniofacial mesenchymal and neural tissues originate from cranial neural crest cells, which migrate and combine with the mesoderm and epithelium to establish facial primordia [2]. Craniofacial malformations significantly impair patients’ quality of life [3,4]. Craniomaxillofacial prosthetic reconstruction provides patients with the potential to restore aesthetics and function, thereby improving patients’ physical and psychological well-being.

Owing to the complexity of the craniomaxillofacial anatomy, the repair of craniomaxillofacial defects confront significant challenges. Autografts are widely regarded as the gold standard. Despite their considerable advantages, they face limitations including restricted donor tissue availability, the requirement for dual surgical sites, potential surgical complications, and donor site morbidity [5,6,7]. Meanwhile, allografts and xenografts carry risks of immune rejection, while synthetic substitutes exhibit inadequate resorption, poor osseointegration, and increased infection susceptibility [8]. These limitations highlight the critical need for innovative clinical strategies to address the thorny problem.

Tissue engineering holds significant promise as an alternative therapeutic approach [9], requiring the combination of stem cells, biomaterials, and growth factors [10]. Stem cells are ideal for regenerative medicine due to their unique capacity for self-renewal and multilineage differentiation to facilitate tissue regeneration. Meanwhile, they create a regenerative microenvironment that promotes wound healing through the secretion of protective factors [11]. The acquisition, isolation, and culture techniques for stem cells have matured, thereby greatly enhancing their utility in regenerative medicine applications [12,13,14].

There are three basic types of stem cells, namely embryonic stem cells (ESCs), induced pluripotent stem cells (iPSCs), and adult stem cells (ASCs). ESCs are classic pluripotent stem cells capable of differentiating into various cell types of the three embryonic germ layers (endoderm, ectoderm, and mesoderm) both in vivo and in vitro, holding great promise for regenerative medicine. However, their use remains controversial due to significant ethical concerns [15]. The emergence of iPSCs has created new opportunities for personalized medicine. These reprogrammed somatic cells exhibit typical ESC characteristics while avoiding ethical issues, making them promising ESC alternatives [16]. However, both ESCs and iPSCs pose safety concerns for cell therapy due to their potent proliferation and differentiation capabilities [17]. Mesenchymal stem cells (MSCs), a subset of ASCs, have shared features with ASCs. In contrast to pluripotent stem cells, MSCs are mesoderm-derived multipotent cells with restricted differentiation potential, primarily committing to mesodermal lineages [18]. The low tumorigenic risk and lack of ethical issues associated with mesenchymal stem cells have led to their extensive application in cell therapy and regenerative medicine [19].

There is no precise definition of MSCs. In 2005, the Mesenchymal and Tissue Stem Cell Committee of the International Society for Cellular Therapy (ISCT) recommended that plastic-adherent cells described as mesenchymal stem cells should be termed multipotent mesenchymal stromal cells, and the term ‘mesenchymal stem cells’ be restricted to cell populations with definitive stem cell properties [20]. Mesenchymal stem cells are a population of mesenchymal stromal cells. Notably, regardless of the nomenclature, the abbreviation ‘MSC’ remains unchanged. In 2006, the ISCT established the minimal criteria for defining MSCs: (1) adherence to plastic under standard culture conditions; (2) differentiation potential into osteogenic, chondrogenic, and adipogenic lineages in vitro; (3) expression of surface markers CD73, CD90, and CD105; and (4) lack of expression of hematopoietic and endothelial markers, including CD34, CD45, CD14, CD11b, CD79a, CD19, and HLA-DR [21]. However, due to the heterogeneity of MSCs across different tissues, some bone marrow mesenchymal stem cells (BM-MSCs) and adipose-derived stem cells (ADSCs) may also express CD34, making it difficult to establish a precise definition for MSCs [22]. Although guidelines recommend alternative terms such as ‘mesenchymal stromal cells’, the term ‘mesenchymal stem cells’ remains prevalent in current research.

Due to their lack of significant ethical problems and that they are safe to obtain, MSCs are widely adopted in regenerative medicine [23]. Initially isolated from bone marrow [24], MSC-like populations have subsequently been isolated and cultivated from adipose tissue [25,26], skeletal muscle tissue [27,28], placenta [29], umbilical cord [30], and amniotic fluid [31]. Although BM-MSCs remain the most prevalently utilized in tissue engineering, there are several constraints in their application, such as low cell production, limited self-renewal and differentiation potential, invasive harvesting procedures, technical complexity, and donor site infection risks [32]. In recent years, dental stem cells (DSCs) have gained increasing attention owing to their similarities to BM-MSCs, abundant content, and minimally invasive isolation procedures [33].

In light of the significance of the craniofacial region and the exigency of repairing congenital defects in craniofacial tissues, along with the superiority of craniofacial-derived MSCs in tissue regeneration, this review concentrates on the application of craniofacial-derived MSCs in treating craniofacial congenital defects and associated challenges in this process.

## 2. Safety and Efficacy of Mesenchymal Stem Cells

Chromosomal aberrations have been identified in the culture of *human* ESCs and iPSCs that increase the malignant potential and undermine the cellular differentiation capacity [34,35,36]. Some researchers propose that specific chromosomal aberrations may exist in *human* ASCs, potentially leading to tumorigenesis. These findings raise significant safety concerns for MSC-based regenerative therapy. A study conducted chromosomal aberration analysis in *human* MSCs, neural stem cells, and hematopoietic stem/progenitor cells. They discovered that the aberration rate of 144 MSC samples from 5 distinct sources was 4%, which indicated potential carcinogenic risk. This study provided evidence for MSC genomic instability, but they did not perform validation tests to evaluate the immortalization and in vivo tumorigenicity of MSCs [34]. In a related study, after a year of continuous in vitro passaging of BM-MSCs from *mice*, the cells acquired unlimited proliferative capacity, lost differentiation potential, and induced neoplasia upon in vivo transplantation [37]. Canine MSCs demonstrated the potential for spontaneous transformation, resulting in osteosarcoma. However, the probability was much lower compared to *mice* [38]. Interestingly, during the continuous passaging of *human* BM-MSCs (hBM-MSCs), only senescence was observed, with no evidence of immortalization [37]. These findings indicate that *human* stem cells possess intricate genomic stabilization mechanisms, making the *human* genome more stable than that of non-primate animals. Tarte et al. [39] revealed chromosomal aberrations in 5 out of 20 in vitro cultured hBM-MSC samples using karyotype analysis. However, these aberrant clones showed no growth advantage during prolonged culture. Furthermore, when injecting chromosomally aberrant MSCs into immunocompromised *mice*, no tumorigenesis was observed. Similarly, dental pulp stem cells (DPSCs) and periodontal ligament stem cells (PDLSCs) did not spontaneously immortalize during prolonged in vitro culture [37]. The above findings challenge the hypothesis that acquiring specific chromosomal aberrations provides a growth advantage to *human* MSCs (hMSCs).

Three subclones exhibiting chromosomal instability were isolated from heterogeneous dental follicle stem cells (DFSCs) during prolonged culture in vitro [40]. Notably, these subclones did not exhibit high levels of tumor markers or induce tumorigenesis in vivo. Individually cultured subclones decreased tumor suppressor p53 expression and developed tumor-associated numerical aneuploidy. However, co-culture conditions restored chromosomal instability and normalized p53 expression, suggesting that the co-culture environment recapitulates the native microenvironment and promotes cellular recovery. These findings indicate that maintaining MSC heterogeneity during in vitro culture can preserve their genetic stability by simulating the native microenvironment.

Although the acquisition of chromosomal aberrations during the culturing process may induce spontaneous immortalization, hMSCs demonstrate positive outcomes without acquiring tumorigenic properties. These findings suggest that hMSCs maintain genomic stability. However, routine monitoring remains crucial during in vitro cultivation to ensure the safe clinical application of hMSCs. Thorough safety assessment is essential prior to the clinical transplantation of MSC-based therapies. Humanized *mouse* models offer valuable preclinical insights by recapitulating the *human* immune microenvironment, thereby providing more physiologically relevant data. These models enable investigation of MSC immunomodulatory properties and facilitate evaluation of therapeutic safety, efficacy, potential toxicity, and tumorigenic risk in vivo [41,42,43]. Repeated intranasal administration of hMSCs to juvenile immunodeficient *mice* revealed no evidence of tumorigenesis or pathological abnormalities in examined tissues. These cumulative findings substantiate the safety of chronic intranasal MSC delivery, positioning this non-invasive approach as a viable candidate for clinical development [44]. However, notable discrepancies persist between humanized *mouse* models and *humans* in terms of both immune system function and organ microenvironment characteristics, necessitating the development of more physiologically relevant models that better recapitulate *human* biology.

## 3. The Senescence of Mesenchymal Stem Cells

Before clinical administration, MSCs have to be expanded many times to obtain a sufficient number. However, many previous studies indicated that passage doubling inevitably incurs cell senescence, which involves lysosomal acidification dysfunction, DNA replication mistakes, telomere attrition, reactive oxygen species (ROS) generation, mitochondrial dysfunction, and epigenetic alterations [45,46,47,48,49,50,51,52].

Lysosomes mainly perform the function of degrading micro-molecules through autophagy and recycling metabolic byproduct, which is closely dependent on the internal pH value, ranging from 4.5 to 5.0 [53]. The abnormal increase in internal pH would weaken lysosomal digestion ability, impair toxic protein clearance, and even contribute to the accumulation of toxic byproducts, which is the main cause influencing MSC aging [54,55]. Excessive ROS is one of the toxic byproducts, and it is also another factor accelerating MSC aging [49]. A pathological dosage of ROS triggers the DNA damage response (DDR), mitochondrial disruption, autophagy inhibition, and telomere attrition. At the same time, all these negative effects of ROS could induce cells to age [56].

DNA damage, including DNA replication mistakes and telomere attrition, is the corollary of MSC passage doubling. Cell cycle arrest was initiated by DDR and manipulated by the p21^CIP1^/p16^INK4A^ signal pathway [51]. p21^CIP1^ and p16^INK4^ decrease the phosphorylation level of RB and then suppress E2F, CDK4, and CDK9, thereby inducing MSC cell cycle arrest and prevent cell proliferating [57,58]. Telomere is a nucleoprotein cap at the end of linear chromosomes, guaranteeing chromosomal stability. Proper telomere length is essential for cell function [59]. However, length shrinkage occurs with MSC division. Telomerase maintains telomere length homeostasis through repairing 5′-TTAGGG repeats, which has been demonstrated to inhibit p53, activate the TGF-β/Smad signal pathway, and induce SIRT1 to combine with telomerase [60,61,62].

As the cellular power house, mitochondria are the major site for aerobic respiration. Mitochondrial malfunction correlates with an imbalanced ratio of ATP/ADP and NAD^+^/NADH; therefore, it could induce cell aging through activating the p53/p21^CIP1/WAF1^ and AMPK/p53 pathways, respectively [63,64]. As previously mentioned, mitochondrial malfunction is a critical component of cell senescence induced by ROS. Abnormally high ROS levels would lead to mitochondrial DNA (mtDNA) damage, which is recognized to be more susceptible to mutation than nuclear DNA [48].

Except for the factors mentioned above, epigenetic regulation, including DNA methylation, histone modification, and chromatin modeling, is another catalyst with higher acceptance. DNA methyltransferases regulated DNA methylation status, in which DNMT1, DNMT3A, and DNMT3B isoforms were involved. Ah-Young, et al. found that DNMT1 and DNMT3B are decreased in hUCB-MSC senescence [65]. Histone lysine acetylation levels played an essential role in chromatin architecture. In general, histone acetylation enabled an open chromatin configuration with active transcription, whereas histone deacetylation lead to heterochromatin, suppressing gene expression and then cell senescence [66]. In addition, it has been verified that histone deacetylases (HDAC) inhibitors could cause MSC cell cycle arrest at G2/M via activating the p21^CIP1/WAF1^ pathway [67]. Chromatin modeling alters the chromatin landscape into an active or suppressed state. Brahma-related gene 1 (BRG1) is a subunit of the ATPase of the SWI/SNF chromatin remodeling complex and is widely considered as an essential regulator of chromatin modulation. Silencing BRG1 and upregulation of NANOG were associated with recruitment of DNMT1; thus, CpG of NANOG is methylated and subsequently suppresses aging [68].

MSC senescence, a multifactor-regulated process compromising therapeutic outcomes, critically diminishes proliferative capacity and functionality. Strategic countermeasures to prevent or delay cellular senescence including the use of antioxidants, small metabolites, and genetic engineering are essential for enhancing MSC-based therapies [45,56,69,70].

## 4. Sources and Characteristics of Craniomaxillofacial-Derived Mesenchymal Stem Cells

### 4.1. Cranial Suture Stem Cells

The understanding of resident stem cells and their niches in craniofacial bones remains limited. The identification of the inter-sutural mesenchyme as a distinct stem cell niche brought suture stem cells (SuSCs) to the attention of the scientific community. Recently isolated SuSCs constitute a heterogeneous population with self-renewal and multilineage differentiation capabilities and play a crucial role in normal cranial growth and development [71]. Previous studies have demonstrated distinct MSC populations within cranial sutures, including Gli1^+^ MSCs [72], Axin2^+^ MSCs [73], Prrx1^+^ MSCs [74], and Ctsk^+^ MSCs [75], all of which contribute significantly to cranial repair and regeneration processes.

### 4.2. Stem Cells Derived from the Oral Cavity

Oral cavity-derived MSCs represent one of several stem cell populations residing in specialized tissues. Since the successful isolation and culture of DPSCs by Gronthos et al. in 2000 [76], various DSCs have been progressively isolated and characterized, including stem cells from *human* exfoliated deciduous teeth (SHED), PDLSCs, stem cells from apical papilla (SCAP), DFSCs, and gingival mesenchymal stem cells (GMSCs) [77]. Current research demonstrates that oral cavity-derived MSCs have undergoing osteogenic, chondrogenic, adipogenic, and neural differentiation capacities under appropriate conditions [78]. Similar to other ASCs, DSCs show significant population heterogeneity. Despite originating from neural crest cells, these cells vary considerably in characteristics, including differences in proliferative capacity, multilineage differentiation potential, and the expression of cell surface receptors and other genes [79].

Most DSCs express characteristic markers such as CD29, CD44, CD73, CD90, and CD105, but lack hematopoietic lineage markers, including CD14, CD34, CD45, and *human* leukocyte antigen HLA-DR. Recent studies have identified STRO-1 and CD146 as specific markers for DSCs [77,78]. Although these markers are also expressed by other MSCs [80], their expression levels are notably higher in DSCs. Table 1 summarizes the expression profiles of common MSC surface markers.

#### 4.2.1. DPSCs

DPSCs were initially isolated from *human* impacted third molars in 2000 and have since been identified as a distinct population of dental mesenchymal stem cells [76]. These cells can also be obtained from premolars removed for orthodontic reasons, exhibiting significant reparative and regenerative potential. DPSCs primarily reside in the dental pulp neurovascular niche, where they express specific vascular-associated antigens and show robust angiogenic capabilities [95]. Although BM-MSCs are more commonly utilized in cell therapy, studies have shown that DPSCs exhibit superior proliferative and differentiative potential [96]. However, it is important to recognize that excessive in vitro expansion can affect the telomere length of DPSCs, resulting in cellular senescence. Therefore, it is recommended to limit the frequency of passaging during the in vitro cultivation of DPSCs [97].

#### 4.2.2. SHED

SHED were successfully isolated in 2003 [98]. Compared to other stem cells, SHED represent the most accessible odontogenic stem cells with minimally invasive procedures during the replacement phase of deciduous teeth [99]. Research has demonstrated that SHED express STRO-1 and CD146, along with neuronal and glial cell markers. SHED possess the capability to differentiate into nerve cells, adipocytes, and odontoblasts in vitro and form bone generate dentin in vivo. Although SHED exhibit greater proliferative capacity and bone induction potential compared to DPSCs, they cannot regenerate the complete dentin–pulp complex [98].

#### 4.2.3. SCAP

SCAP were initially isolated from the apical papilla of unerupted third molars in 2006 [100]. These cells are prevalent in the apical regions of teeth and exhibit typical MSC characteristics. SCAP demonstrate high expression levels of survivin and telomerase, contributing to their enhanced proliferative capacity. Consequently, SCAP possess robust proliferative capacity and significant osteogenic differentiation potential, but with limited adipogenic differentiation potential. These cells can form dentin structures in vivo and exhibit odontoblastic differentiation capability [100,101]. SCAP exhibit higher proliferation rates, increased mineralization potential, and enhanced migratory ability compared to DPSCs [102]. Isolated SCAP can be cryopreserved in liquid nitrogen without compromising their biological properties or immunophenotype [103]. Notably, during pulpitis and periapical inflammation, the root apex is frequently preserved, allowing SCAP to retain cell viability and stemness [90]. This preservation supports continued root development following treatment for inflammation-induced developmental arrest.

#### 4.2.4. PDLSCs

The periodontal ligament is a fibrous connective tissue that anchors teeth to the alveolar bone, primarily including fibers, cells, and extracellular matrix (ECM), with neurovascular bundles providing nutrient supply [104]. PDLSCs were first isolated and characterized from impacted third molars in 2004 [89] and can also be harvested from orthodontically extracted teeth [105]. PDLSCs comprise approximately 95% MSCs and 5% neural crest stem cells. Furthermore, PDLSCs express surface markers similar to BM-MSCs and DPSCs [106] and have been demonstrated to generate cementum/periodontal ligament (PDL)-like tissues in vivo [89], establishing them as an excellent cell source for periodontal tissue regeneration. Additionally, unlike other MSCs, PDLSCs exhibit high expression levels of the tendon/ligament-associated transcription factors, which make PDL withstand mechanical stress [89].

Previous studies demonstrated that PDLSCs exhibit superior proliferative and neurogenic differentiation capacities compared to DPSCs [107,108]. Conversely, single-cell RNA sequencing analysis revealed that PDLSCs showed lower proliferative potential and stemness relative to DPSCs [109]. This discrepancy may be attributed to the methodological differences. The latter utilized single-cell and large-scale RNA sequencing for comparative analysis, while the former focused on the specific genes associated with stemness.

#### 4.2.5. GMSCs

In 2009, Zhang et al. [110] successfully isolated a stem cell population from gingival tissues that was characterized as GMSCs. These cells can be easily obtained during routine dental procedures. Approximately 90% of GMSCs originate from cranial neural crest cells, while the remaining 10% are derived from the mesoderm [93]. A study successfully isolated STRO-1-positive (MACS^+^) and STRO-1-negative (MACS^−^) cell populations from *human* free gingival margin using STRO-1/magnetic activated cell sorting (MACS). Comparative analysis revealed that MACS^+^ cells exhibited enhanced stem/progenitor cell characteristics and greater multilineage differentiation potential relative to their MACS^−^ counterparts. These findings establish STRO-1 as a positive selection marker for GMSC isolation [111]. Another study excluded CD146 as a specific MSC surface marker for isolating or enriching MSC populations from the total gingival fibroblast pool [92]. GMSCs possess superior proliferative capacity and colony-forming unit efficiency compared to PDLSCs and DPSCs [112]. Furthermore, GMSCs not only modulate the phenotype of innate and adaptive immune cells, but also activate them [113]. Unlike PDLSCs, GMSCs maintain stable growth patterns and immunomodulatory characteristics regardless of donor’s age. GMSCs also demonstrated superior preservation of osteogenic capacity, exhibiting fewer inflammation-associated alterations in both in vitro differentiation and in vivo bone formation [114]. Notably, their neurogenesis capacity remains preserved in vitro, indicating that GMSCs possess significant potential for neuronal regeneration and nerve repair applications [115]. Unlike other MSCs, GMSCs offer higher accessibility and rapid scalability for clinical applications [116]. Critically, they retain MSC properties, phenotypic stability, and telomerase activity during prolonged culture, with no observed tumorigenicity [117]. Thus, GMSCs offer distinct therapeutic advantages in craniofacial regeneration due to their easy isolation, stable phenotype, preserved genomic stability, robust expansion potential, and maintained telomerase activity throughout prolonged cultivation.

#### 4.2.6. DFSCs

The dental follicle can develop into cementum, periodontium, and alveolar bone. DFSCs isolated from the developing third molar follicle demonstrate the capacity to differentiate into osteoblasts and odontoblasts [118]. *Human* DFSCs were first isolated and characterized in 2005 [119]. These cells highly express stemness markers, such as Stro-1, Nestin, Notch-1, and Oct4. DFSCs represent a promising stem cell source for bone regeneration because of strong osteogenic properties [120].

**Figure 1:**
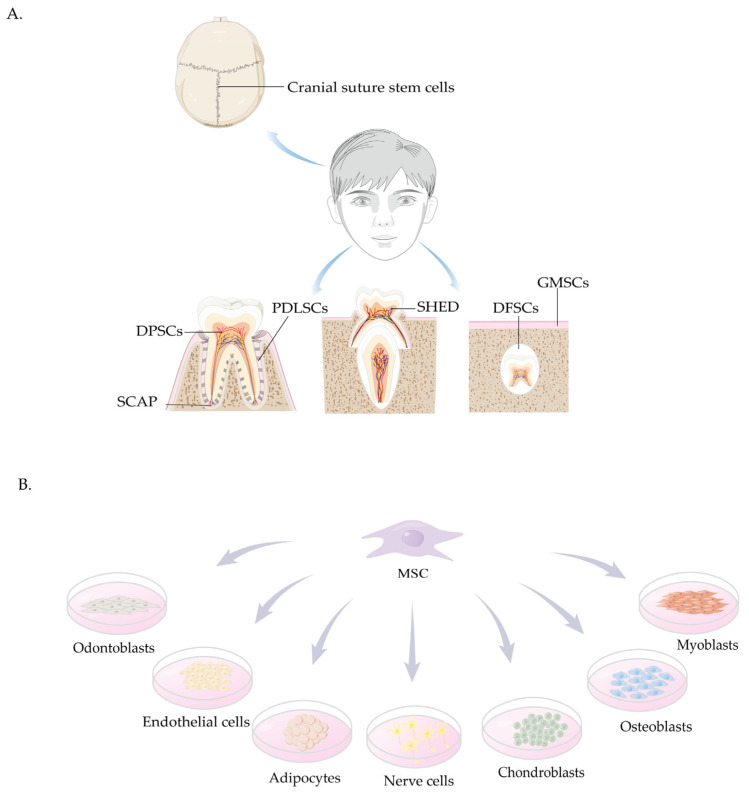
Craniomaxillofacial-derived mesenchymal stem cells (MSCs). (**A**) Major craniomaxillofacial-derived MSCs include cranial suture stem cells, dental pulp stem cells (DPSCs), stem cells from apical papilla (SCAP), periodontal ligament stem cells (PDLSCs), stem cells from *human* exfoliated deciduous teeth (SHED), gingival mesenchymal stem cells (GMSCs), and dental follicle stem cells (DFSCs). Color coding represents anatomical structures in the dental pulp: red lines (arteries), blue lines (veins), and yellow lines (nerves). (**B**) MSCs demonstrate multilineage differentiation potential, giving rise to myoblasts, osteoblasts, chondroblasts, nerve cells, adipocytes, endothelial cells, and odontoblasts.

The craniomaxillofacial-derived MSCs are illustrated in Figure 1.

### 4.3. Comparison of Craniomaxillofacial-Derived Mesenchymal Stem Cells and Other Tissue-Specific Stem Cell Populations

DSCs can be easily isolated and extracted from medical waste generated during routine dental procedures. Compared to BM-MSCs, DSCs have superior accessibility, reduced invasiveness, and eliminate additional patient risk. These cells are abundantly available and suitable for long-term storage in future utilization. Moreover, unlike ESCs, they avoid safety and ethical concerns [33,80].

**Figure 2:**
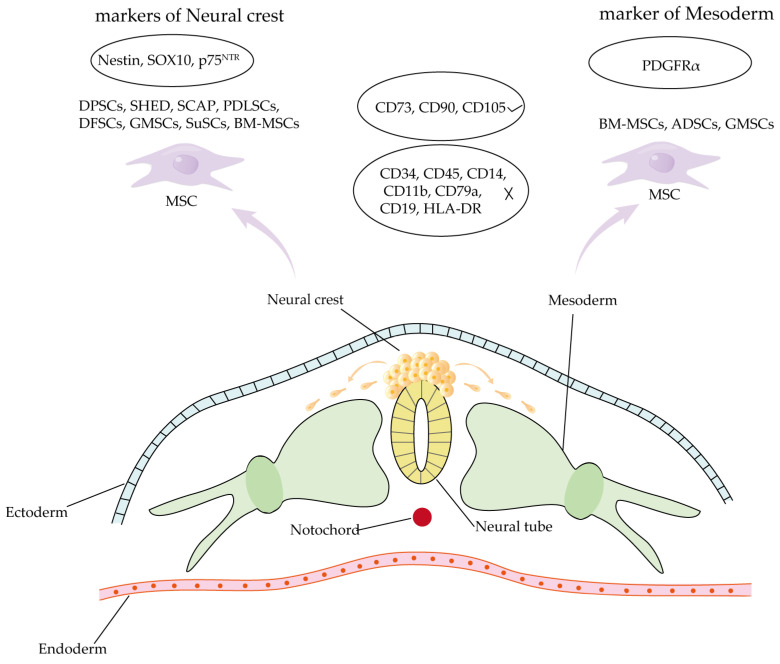
Origins of MSCs. MSCs are derived from both neural crest and mesodermal lineages. The majority of craniofacial-derived MSCs and a subset of BM-MSCs originate from neural crest progenitors and express characteristic neural crest markers (e.g., Nestin, SOX10, p75^NTR^). In contrast, BM-MSCs, ADSCs, and a minor population of GMSCs are of mesodermal origin, exhibiting typical mesodermal markers (e.g., PDGFRα).

Tissue-specific MSCs are widely distributed in various mesoderm-derived organs, whereas DSCs primarily originate from the neural crest (Figure 2) [93,121,122,123,124,125,126,127,128]. Despite their distinct origins, both tissue-specific MSCs and DSCs exhibit similarities in fundamental characteristics, including self-renewal capacity, multilineage differentiation potential, immunomodulatory functions, and expression of MSC-associated surface markers [77]. However, compared to BM-MSCs, DSCs exhibit enhanced odontogenic differentiation potential but reduced capacity for osteogenic, chondrogenic, and adipogenic differentiation [80]. During in vitro differentiation of BM-MSCs, umbilical cord mesenchymal stem cells, ADSCs, DFSCs, DPSCs, PDLSCs, GMSCs, BM-MSCs, and ADSCs exhibited the highest osteogenic differentiation efficiency. PDLSCs and DFSCs showed moderate potential, while GMSCs and umbilical cord mesenchymal stem cells demonstrated the lowest osteogenic capacity. Under serum-free culture conditions, DPSCs and PDLSCs displayed notably enhanced the anti-apoptotic capability [129]. The osteogenic differentiation capacity of PDLSCs was markedly superior to that of DPSCs and SHEDs, as demonstrated by both functional assays and gene expression profiling [130]. Compared to SHEDs, DPSCs presented higher levels of osteogenic and odontogenic differentiation, which indicated that the latter was inclined to have the phenotype of odontoblasts [88]. At the same time, scRNA-seq analysis in 2024 showed that DPSCs had more “stem-like” cells than PDLSCs [109]. However, SCAPs showed a greater proliferation efficiency and mineralization ability in vitro than DPSCs [102]. Stem cells derived from the oral cavity exhibit distinct immunomodulatory properties. Compared to alveolar bone-derived MSCs (aBMSCs) and GMSCs, DPSCs demonstrate significantly less secretion of osteopontin and weaker immunosuppressive effects on THP-1 monocytic cells [131].

The most significant characteristic of stem cells lies in their exceptional in vitro proliferation capacity and multilineage differentiation potential, which are crucial for the application of stem cell-based tissue engineering and regenerative therapies. Research has demonstrated that DSCs exhibit superior proliferative capabilities compared to BM-MSCs and ADSCs [132].

Given that DSCs are derived from the embryonic neural crest, they not only possess the typical MSCs characteristics but also exhibit neurogenic properties similar to those of neural crest-derived stem cells. The neurological differentiation capacity varies among different types of dental stem cells [133,134]. Research has indicated that *human* DPSCs outperform in promoting axon growth and the establishment of neuronal networks compared to *human* bone marrow stromal cells (hBMSCs) [134].

## 5. Applications of Stem Cell Therapy in Congenital Craniofacial Defects

Craniofacial development begins during early embryogenesis. Disruptions in this process can lead to corresponding malformations, which may arise from genetic mutations, chromosomal abnormalities, or environmental factors [135]. Congenital craniofacial defects comprise a spectrum of abnormalities. This review primarily focuses on cleft lip and palate, craniosynostosis, and tooth agenesis.

### 5.1. Cleft Lip and Palate

Cleft lip and palate represent the most common congenital craniofacial anomaly [136], primarily caused by developmental disruptions in the soft and hard tissues surrounding the oral and face regions. This condition may present as unilaterally or bilaterally [137].

Cleft lip and palate may occur as part of syndrome or as non-syndromic conditions, with the latter being more clinically prevalent [99,138]. Non-syndromic orofacial clefts include cleft palate and cleft lip with or without cleft palate (CL/P). Syndromic forms following Mendelian inheritance patterns are associated with definitive genetic pathogenic factors [137]. Non-syndromic cases result from complex interactions among genetic, epigenetic, and environmental factors [53]. To date, over 45 common variants associated non-syndromic CL/P have been identified in genome-wide association studies [139].

The management of CL/P primarily involves surgical intervention, requiring a multidisciplinary approach with multiple procedures throughout the patient’s lifespan to address tissue deficiencies, malocclusion, speech and hearing impairments, and aesthetic concerns. Given the insufficient tissues in the lesion area, bone augmentation procedures frequently require supplementation from alternative anatomical sites. The procurement of autologous bone is the most prevalent option [140]. Owing to the necessity for additional surgeries and infection risk, tissue engineering offers novel solutions for the management of CL/P. DSCs exhibit a high capacity for osteogenic differentiation [141,142]. Regrettably, because animal models of cleft palate often experience feeding difficulties and other developmental issues post-birth [143,144], research focusing on bone reconstruction and tissue repair remains limited [99]. Numerous scholars have conducted extensive research on tissue repair and regeneration by creating alveolar bone or cranial bone defects in animals to simulate alveolar clefts or cleft palates. The most frequently utilized animal models include *mice*, *rats*, *rabbits*, and *minipigs* [145]. Since cranial bone defects in *mice* are typically larger than alveolar bone defects, they more closely resemble the dimensions of *human* alveolar cleft defects. Therefore, the majority of studies have focused on inducing cranial bone defects to simulate *human* alveolar clefts [146]. Studies have demonstrated that MSCs possess significant potential to regenerate bone tissue at the defect site [147,148]. Clinical trials treating cleft lip and palate with MSCs are presented in Table 2. The following section will primarily focus on the application of oral cavity-derived stem cells in repairing cleft lip and palate using animal models.

#### 5.1.1. DPSCs in Cleft Lip and Palate Repair

DPSCs, transplanted in conjunction with a dense collagen gel scaffold into a critical-sized calvarial defect in *rats*, enhanced the osteoconductive properties of the scaffold. This combination facilitated the migration and attachment of endogenous osteoprogenitor cells, thus promoting bone healing [149]. This finding suggests that the combination of DPSCs with an appropriate physiological scaffold represents a promising approach for improving bone defect repair. Additionally, when DPSCs and Matrigel were transplanted into a maxillary alveolar defect in *rats*, researchers observed the formation of trabecular bone structures at the defect site within 14 days post-surgery. At the same time, the treatment promoted osteoblast proliferation and enhanced the osteogenic differentiation capacity of DPSCs, thereby improving bone regeneration [150].

#### 5.1.2. SHED in Cleft Lip and Palate Repair

SHED isolation is devoid of adverse health consequences [151]. Studies in animal models of cleft lip and palate have revealed that SHED exhibits superior osteogenic properties. Lee et al. [152] demonstrated that *human* SHED cell sheets could be successfully transplanted into the *mice* palatal bone, maintaining robust osteogenic potential, and it was observed that SHED cell sheets possessed higher osteogenic capability compared to hMSCs cell sheets. The combination of SHED and scaffolds exhibits comparable regenerative potential to autologous bone transplantation for repairing edentulous maxillary sockets [151]. Prior studies indicate that MSCs promote tissue repair and regeneration through paracrine secretion of cytokines and growth factors [33]. Research on SHED conditioned medium (SHED-CM) demonstrated its superior therapeutic efficacy compared to SHED in repairing cranial defects in immunodeficient *mice* [153].

Several clinical trials have evaluated stem cell efficacy in bone regeneration for patients with cleft lip and palate. One study isolated SHED from the six patients with unilateral alveolar clefts, and subsequently transplanted SHED with Bio-Oss Collagen into alveolar defects of the patients. The findings indicated that all patients who underwent tissue-engineered transplantation demonstrated alveolar bone healing comparable to iliac crest bone grafting [154]. These findings position SHED and SHED-CM as promising alternatives for cleft palate and alveolar defect reconstruction.

#### 5.1.3. Additional Stem Cell Populations Derived from the Oral Cavity

GMSCs were transplanted with the self-assembling hydrogel scaffold PuraMatrix™ (PM) and/or bone morphogenetic protein 2 (BMP2) into the maxillary alveolar defect sites of immunodeficient *mice*, showing significant enhanced bone regeneration at 4 and 8 weeks post-surgery. Compared to the single material control group, the PM/GMSCs/BMP2 combination exhibited superior bone regeneration efficiency [155]. This discovery offers a novel source of stem cells for cleft-related tissue repair. However, this study lacked an autologous iliac bone graft control group. Furthermore, the study utilized single donor samples, requiring further validation of GMSCs’ bone regeneration capacity.

Zhao et al. [105] successfully isolated and extracted *human* PDLSCs from healthy premolars and subsequently isolated PDLSC-derived extracellular vesicles (P-EVs) during in vitro culture. When immobilized in Matrigel and transplanted into *rat* cranial defect, P-EVs significantly enhanced cell proliferation and bone repair efficacy after 10 weeks, suggesting a promising cell-free therapeutic strategy. However, the regenerative capacity and differentiation potential of PDLSCs may be influenced by the donor’s age. Future investigations should focus on this critical factor.

Cells isolated from orbicularis oris muscles during cleft lip and palate repair surgeries were confirmed as MSCs using surface antigen and hematopoietic marker analysis [156]. When transplanted into cranial defects of immunodeficient *rats*, these cells demonstrated bone regeneration, suggesting their potential as a therapeutic source for cleft lip and palate treatment.

Oral cavity-derived stem cells have exhibited significant bone regeneration capabilities in cleft lip and palate models. However, due to a paucity of animal models for congenital cleft lip and palate and limited clinical trials, it remains undetermined whether these stem cells can achieve comparable outcomes across various repairment approaches, warranting further investigation.

### 5.2. Craniosynostosis (CS)

Craniosynostosis denotes the premature fusion of one or more cranial sutures [157], representing the second most common craniofacial anomaly following cleft lip and palate. Cranial sutures, the fibrocellular junctions connecting skulls, primarily consist of metopic, coronal, sagittal, and lambdoid sutures [158]. These sutures originate from MSCs derived from the neural crest and mesoderm. As growth centers, cranial sutures enable coordinated skull expansion during brain development. Moreover, they accommodate necessary skull deformation during childbirth and facilitate postpartum restoration of the cranial vault to its original configuration [159,160]. However, premature bone fusion, often resulting from precocious puberty, localized bone growth defects, or genetic factors leading to cranial suture stem cell dysfunction, disrupts continuous postnatal growth. This premature closure can result in severe complications, including microcephaly, facial asymmetry, intracranial hypertension, impaired cerebral blood flow, and severe neurological damage, all of which significantly affect normal growth and development [158,161,162]. Craniosynostosis presents as either syndromic (approximately 20%) or non-syndromic (approximately 80%). Recent research has identified over 100 *human* gene mutations linked to syndromic craniosynostosis [163]. The most prevalent forms of syndromic craniosynostosis include Muenke type (*FGFR3* mutations), Saethre-Chotzen type (*TWIST1* mutations), Crouzon type (*FGFR2* mutations), Pfeiffer type (*FGFR1* and *FGFR2* mutations), and Apert type (*FGFR2* mutations) [164,165].

Currently, the primary approach to treating craniosynostosis involves surgical cranial vault reconstruction, requiring extensive skull dissection from the underlying dura mater. Despite low immediate complication rates, the high incidence of restenosis leads to significant long-term risks [163]. Exploring alternative therapeutic strategies for craniosynostosis remains an urgent priority.

Implanting Gli1^+^ MSCs and modified GelMA hydrogel into the cranial sutures of craniosynostosis *Twist1^+/−^ mice* promotes suture regeneration, corrects skull deformities, restores intracranial pressure (ICP), and improves neurocognitive function [166]. This minimally invasive approach offers an effective regenerative treatment for craniosynostosis. However, since Twist1 is not expressed in adult *mouse* brains but is expressed in *human* brains during fetal and adulthood phases, further studies are needed to validate its clinical applicability. Maruyama et al. [167] demonstrated the presence of Axin2^+^ stem cells within the cranial sutures. These cells maintain the SuSC characteristics during in vitro culture. Long-term observations confirmed their self-renewal capacity. When transplanted ectopically into the renal capsule of *mice*, these cells formed intramembranous bone tissue similar to the skull, but not cranial suture-like structures. This is likely due to the renal capsule environment’s inability to replicate the cranial suture niche. This study provides new cell source for craniosynostosis treatment. A novel stem cell population, Ddr2^+^ MSCs, was identified in cranial sutures [168]. The absence of Ctsk^+^ MSCs leads to Ddr2^+^ MSCs proliferation and activation, promoting endochondral osteogenesis and resulting in craniosynostosis. Inhibiting Ddr2^+^ MSCs may offer an effective therapeutic approach for premature suture closure. However, isolating and culturing *human* SuSCs and maintaining niche cells remain challenging. Further investigation is imperative to optimize techniques and prevent suture restenosis and premature closure.

### 5.3. Tooth Agenesis

Tooth agenesis refers to the absence of one or more teeth during the developmental stage. This condition affects both deciduous and permanent dentitions, with higher prevalence in the latter. The most frequently impacted teeth are the third molar, second premolar, and maxillary lateral incisor. Tooth agenesis has a multifaceted etiology, including genetic and environmental factors. This condition can occur independently or as part of syndromes, often associated with other ectodermal anomalies, such as cleft lip and palate [169]. Tooth agenesis exhibits genetic heterogeneity, encompassing autosomal dominant, autosomal recessive, and X-linked inheritance patterns [170]. Recent studies have identified several genes associated with tooth agenesis, including *MSX1*, *PAX9*, *AXIN2*, *WNT10A*, *EDA*, and *RUNX2* [171,172].

**Figure 3:**
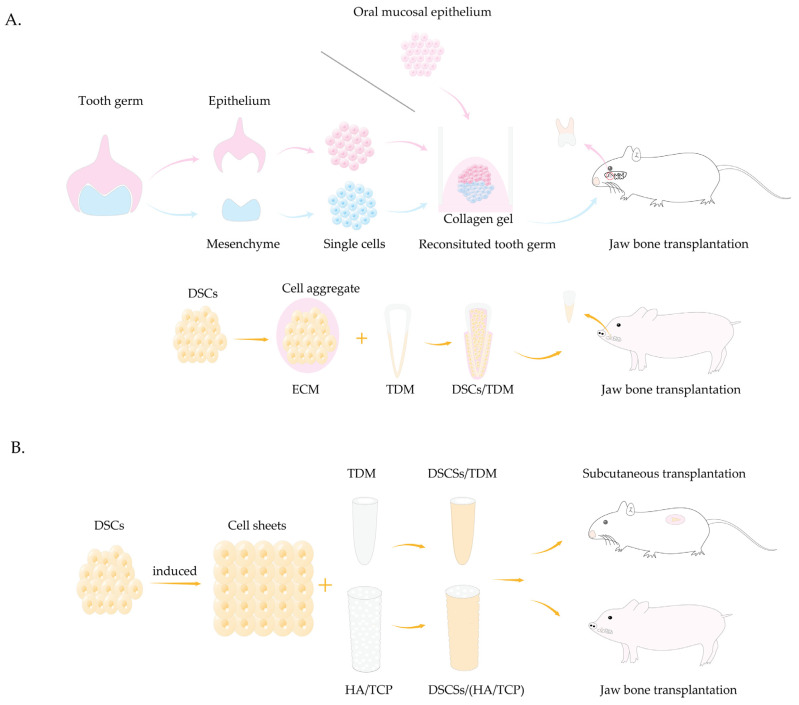
Schematic diagram of tooth regeneration construction. (**A**) Whole tooth regeneration. Dissociate bell-stage tooth germ epithelium and mesenchyme into single cells and reconstruct at high density. Transplant reconstructed cells into *mouse* jaw defects for whole tooth regeneration. The oral mucosal epithelium can replace the tooth germ epithelium in this process. Constructing DSC aggregates combined with TDM scaffolds for functional tooth regeneration in *minipig* alveolar sockets. (**B**) Biological root regeneration. Generate DSC-derived cell sheets and combine with TDM or HA/TCP scaffolds. Transplant them into *mouse* subcutaneous tissue or *minipig* jaw incisor defects for root reconstruction. DSCs, dental stem cells; TDM, dentin matrix; HA/TCP, hydroxyapatite tricalcium phosphate.

Congenital tooth agenesis significantly impacts *human* health by impairing chewing function, causing speech disorders and aesthetic issues, thereby substantially reducing patients’ quality of life [173]. The primary treatment options are dental implants and dentures [174]. Although these methods can partially restore dental function and appearance, they still limited compared to natural teeth. Dental implants lack physiological mobility and cannot adequately respond to external stimuli. Additionally, their maintenance and repair can impose substantial economic burdens. Moreover, complete or partial dentures have limited effectiveness in restoring chewing function and may cause alveolar bone resorption. Several methods for tooth regeneration are introduced (Figure 3).

Teeth development occurs through interactions between oral epithelial and neural crest-derived mesenchymal cells [175]. Thus, tooth regeneration research centers on regenerating these cell types and their interactions. A novel tooth germ culture method was developed by dissociating the bell-stage tooth germ epithelial and mesenchymal tissues into single cells and injecting them into high density collagen gel [176]. This approach successfully reconstructed tooth germs, generating whole teeth in vitro and enabling bioengineered tooth development in *mouse* mandibular defects and renal capsules. These bioengineered teeth exhibit all the structural characteristics of natural teeth, offering a groundbreaking strategy for whole tooth regeneration. Another study confirmed that bioengineered teeth not only restored normal masticatory function but also successfully reconstructed functional periodontal ligament (PDL) capable of responding to mechanical stress and external harmful stimuli [177]. Several studies have employed a similar approach and successfully achieved whole tooth regeneration in the jaws of *minipigs* [178]. However, both methods rely on the acquisition of embryonic tooth germs and ESCs, along with large cell quantities [179], posing significant ethical and technical challenges for *human* applications.

The oral mucosal epithelium can serve as a potential dental epithelium source. According to the study by Nakagawa et al. [180], the palatal mucosal epithelium of young *mice* could differentiate into dental epithelium and subsequently formed enamel. When combined with dental mesenchymal cells and transplanted, it generated tooth structure. These findings support epithelium and mesenchyme reconstitution through cell reaggregation. The process mimics embryonic development by culturing bioengineered tooth germs in vitro, rather than achieving whole tooth regeneration through alternative stem cell approaches.

Mesenchymal concentration critically influences early tooth formation, determining and guiding tooth morphogenesis. Some studies have constructed DSC aggregates by integrating DSCs and ECM to maintain cell stemness and stability [175,181,182,183]. SHED aggregates combined with treated dentin matrix (TDM) scaffolds to re-establish the tooth development niche and form a structure similar to natural teeth when implanted in *minipig* alveolar sockets [184]. Given the challenges of whole tooth regeneration, root regeneration offers a more viable approach for dental restoration. After developing biological roots, synthetic crowns can effectively restore the tooth shape and function. A study integrated dental follicle cells (DFCs) and SHED with TDM scaffold and transplanted them into nude *mouse* subcutaneous areas and Sprague-Dawley (SD) *rat* jawbones. The results demonstrated the formation of periodontal ligament-like fibrous tissue, dentin, blood vessels, and nerve fibers in both groups [185]. Someone used SCAP and PDLSCs in combination with hydroxyapatite tricalcium phosphate (HA/TCP) scaffolds and successfully reconstruct biological roots in *minipigs* mandible defects [100]. However, it has also been shown that HA/TCP stents have poor compressive properties as well as strength, limiting their utilization [186]. DPSCs also show potential for biological root regeneration through tissue engineering [187].

With advancing tooth organoid research [188,189,190,191], epithelial tooth organoids from neonatal *mice* incisors and molars differentiated into ameloblast-like cells both in vivo and in vitro [192]. Co-culturing DPSCs with gingival keratinocytes formed epithelial sheaths similar to that during tooth development [193]. Although in vivo whole tooth regeneration unverified, they provide a promising direction for future tooth regeneration research.

The shift from experimental to clinical stages in tooth regeneration technology faces significant challenges. Though the concept of whole tooth regeneration is attractive, practical implementation encounters various obstacles, including dental tissue complexity and physiological functions restoration. Obtaining epithelial and mesenchymal cells from embryonic tooth germs also presents major difficulties. Future research should prioritize ASCs or iPSCs as alternatives to embryonic tooth germs in reconstructing tooth functions. Although biological root development has advanced, challenges persist in crown support, dentin matrix sourcing, and scaffold effectiveness. Addressing incomplete tooth germ development, often due to genetic factors blocking critical developmental pathways, requires further investigation into precise regulatory mechanisms to ensure the successful progression of tooth regeneration. The tooth organoids represent a promising future research direction.

## 6. Therapeutic Limitations of Mesenchymal Stem Cells in Congenital Defects

**Figure 4:**
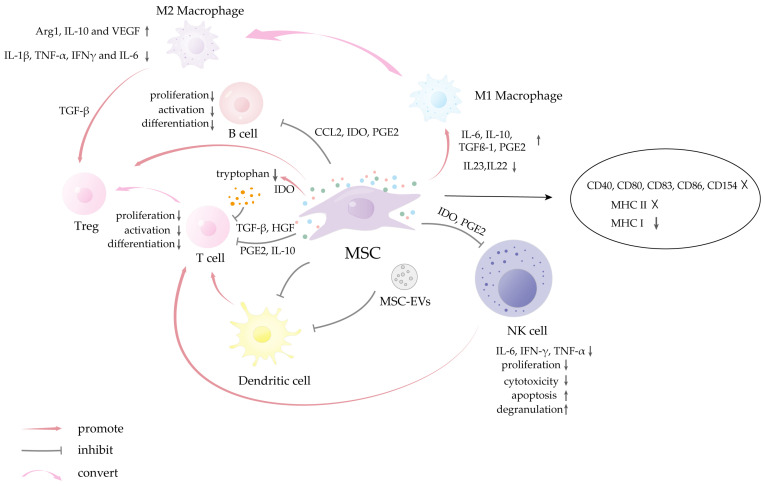
Mechanisms of MSC-mediated immune evasion. MSCs show low immunogenicity due to absence of co-stimulatory molecules/MHC II and low MHC I expression. They modulate immunity by direct contact and paracrine signaling, inhibiting NK cells, DCs, T cells, and B cells as well as inducing M2 macrophages polarization and Treg cells generation. This process requires multiple cytokines and chemokines. PGE2: prostaglandin E2; IDO: indoleamine-pyrrole 2,3-dioxygenase; IFN-γ: interferon-γ; TNF-α: tumor necrosis factor-α; TGF-β: transforming growth factor-β; IL: interleukin; CCL-2: C-C motif chemokine ligand 2; Arg1, arginase 1; VEGF, vascular endothelial growth factor; HGF, hepatocyte growth factor.

For congenital craniofacial defects resulting from genetic abnormalities, stem cell-based regenerative therapy faces significant challenges. Since autologous stem cells retain genetic defects, they are not suitable for direct therapeutic use. Well-characterized allogeneic stem cells have emerged as a promising alternative. However, potential immune rejection due to HLA mismatch poses safety concerns for allogeneic cell therapy. Of note, MSCs appear to avoid this immune rejection (Figure 4).

Generally speaking, the immune rejection response of the host to foreign cells or tissues is mainly through T cell recognition of allogeneic antigen receptors, such as major histocompatibility complex (MHC)/HLA antigens. This recognition triggers T cell-mediated immune responses against allogeneic donor epitopes or MHC complexes to have an immune rejection response, involving both innate and adaptive immune system [194].

Due to the absence of MHC II and classical co-stimulatory molecules such as CD40, CD80, CD83, CD86, and CD154, MSCs exhibit low immunogenicity. Expression levels of MHC I and antigen processing machinery are reduced in these cells [195]. These properties enable MSCs to evade immune detection effectively. MSCs mediate immunomodulation through intercellular contact and paracrine signaling with immune cells. They can directly inhibit the activation and proliferation of NK cells [196]. MSCs also can induce NK cells apoptosis and suppress pro-inflammatory cytokine production such as IL-6, IFN-γ, and TNF-α [197,198]. MSCs promote pro-inflammatory M1 macrophages converting to anti-inflammatory M2 macrophages via prostaglandin E2 (PGE2) [199,200,201]. MSCs inhibit T and B cell proliferation, activation, and differentiation [202,203,204,205]. MSCs also suppress dendritic cell (DC) differentiation, maturation, and antigen-presenting capacity, thereby inhibiting T cell activation. Reis et al. [206] discovered that co-cultures of MSC-derived extracellular vesicles (MSC-EVs) in T cells and monocyte-derived dendritic cells (mDCs) predominantly target DCs. They impair antigen uptake by immature DCs, inhibit DC maturation, and hinder DC migration through the suppression of CCR7 expression, thus exerting immunosuppressive effects.

Immunosuppression can also result from tryptophan depletion and the local accumulation of tryptophan metabolites. MSCs create an inhibitory microenvironment by producing prostaglandins and interleukin-10, as well as expressing indoleamine 2,3-dioxygenase (IDO) to catalyze tryptophan degradation, thereby suppressing the immune response [207].

Although MSCs exhibit potent immunosuppressive capabilities in vitro, some studies have revealed that allogeneic MSCs display limited immunosuppressive effects in vivo and may trigger immune memory, making it challenging to sustain their efficacy over extended periods following infusion in *humans* [208]. To overcome this limitation, clinical trials frequently employ immunosuppressive drugs and repeat MSC transplantation [209], which may bring new safety problems to patients.

Several studies have utilized gene editing techniques to mask HLA and NK cell inhibitory factors to diminish the immunogenicity of MSCs. However, complete ablation of HLA through *B2M* gene knockout may induce NK cell-mediated cytotoxicity, highlighting the necessity for concurrent modulation of both HLA and NK cell inhibitory factors [210,211]. It is feasible to perform multiple gene edits to introduce NK cell inhibitory molecules while preserving only the classical HLA-I, and such extensive gene editing may lead to genomic instability and increase cytotoxicity. Wang et al. [209] utilized an epigenetic editing approach, employing the CRISPR system in hMSCs to target a specific site within intron 1 of the *B2M* gene. This strategy effectively inhibited B2M and HLA-I expression. They constructed a ‘goldilocks-level of β2m expression on MSCs’ (GLOBES), where B2M and HLA-I expression were reduced by more than half but not entirely eliminated. GLOBES effectively evaded the immune responses of allogeneic T cells and NK cells in vitro. GLOBES exhibited prolonged survival without immune activation in humanized *mice*. This approach avoids genomic instability associated with multiple gene edits and additional immunosuppression, offering a crucial strategy for reducing MSC immunogenicity and extending their functional duration in an allogeneic setting. Given the potential risks of off-target editing and viral genome integration inherent in current CRISPR systems, further research is warranted.

## 7. Conclusions and Prospect

The interchangeability use of mesenchymal stem cells and mesenchymal stromal cells remains controversial, although both of them are abbreviated as MSCs. Currently, there are no specific biomarkers capable of reliably discriminating mesenchymal stromal cells from mesenchymal stem cell populations. In 2019, International Society for Cell & Gene Therapy (ISCT^®^) Mesenchymal Stromal Cell committee recommended to maintain the ‘MSC’ designation for mesenchymal stromal cells, while stipulating three critical requirements: (1) mandatory specification of cellular source; (2) restriction of the term ‘mesenchymal stem cells’ exclusively to cases demonstrating rigorous in vitro evidence of self-renewal capacity and multipotent differentiation potential; (23) comprehensive characterization of mesenchymal stromal cells, such as immunomodulation and paracrine functions, through a robust matrix of functional assays with appropriate controls [212]. In 2025, single-cell RNA sequencing (scRNA-seq) and pseudo-time trajectory analysis clearly distinguished mesenchymal stromal cells from stem cells. The researchers found that mesenchymal stromal cells do not express eight stem cell-associated genes (*SOX2*, *NANOG*, *POU5F1*, *SFRP2*, *DPPA4*, *SALL4*, *ZFP42*, and *MYCN*) that are critical for self-renewal and differentiation, while stem cells lack expression of five functional genes (*TMEM119*, *FBLN5*, *KCNK2*, *CLDN11*, and *DKK1*) characteristic of mesenchymal stromal cells [213]. Based on the ISCT standards for mesenchymal stromal cells, Pinkhasov et al. [214] identified ex vivo-expanded lamina propria cells of either the lining or the masticatory oral mucosa as mesenchymal stromal cells. ScRNA-seq analysis classified these cells into three subpopulations: fibroblasts, smooth muscle cells, and mesenchymal stem cells. Notably, all cell populations expressed canonical mesenchymal stromal cell surface markers. Compared to other clusters, mesenchymal stem cells exhibited high expression levels of stemness-associated genes (e.g., *NES*, *PSMD2*, *PSME2*, *PSMC3*, and *DNMT1*) and cell cycle-related genes (*CENPF*, *PTTG1*, *CCNB1*, and *TOP2A*), suggesting their proliferative and undifferentiated state. These findings demonstrated that the interchangeable use of the terms ‘mesenchymal stem cells’ and ‘mesenchymal stromal cells’ is biologically inaccurate, underscoring the need to redefine their classification criteria. Recently, the US FDA approved the world’s first clinical trial explicitly designated as a mesenchymal stromal cell therapy, marking a shift from unproven stem cell therapies to true mesenchymal stromal cell therapies [215]. Considering the prevailing usage in current research literature, we have chosen to use ‘mesenchymal stem cells’ (MSCs) throughout this review to align with the terminology still most commonly used in the literature we cite.

Craniofacial-derived MSCs, sharing the same embryonic origins with craniofacial tissues, possess intrinsic advantages for treating craniomaxillofacial defects. MSCs show broad research applications in craniomaxillofacial medicine, encompassing congenital malformations, tumors, trauma, and temporomandibular joints disorders [216,217,218]. However, several clinical challenges remain, including immunogenicity, long-term safety, and therapeutic efficacy, as well as the limitations in treating maxillofacial defects caused by genetic mutations with genetically defective autologous stem cells. Senescence of MSCs significantly compromises their therapeutic efficacy. Further investigation into therapeutic mechanisms is essential to improve safety and efficacy.

Congenital malformations resulting from genetic factors can create abnormal microenvironments [219]. The capacity of implanted stem cells to effectively promote tissue repair and regeneration in these microenvironments requires further investigation.

Craniofacial defects frequently co-occur with dental anomalies [220]. Tooth regeneration remains a significant challenge. Current tooth regeneration strategies primarily utilize embryonic tooth germs or combine MSCs with scaffolds. Tooth organoids represent a promising research direction.

Overall, craniofacial-derived MSCs show promise for reconstructing craniofacial congenital defects. Further research is needed to understand their biological characteristics and microenvironment interactions. Comprehensive exploration and validation through fundamental research, technological advancement, and clinical trials is essential for widespread clinical application.

## Figures and Tables

**Table 1:** Expression of surface markers in MSCs.

MSC Type	Markers	References
BM-MSCs	CD73, CD90, CD105, CD146, CD29, CD44, OCT4, Nanog, STRO-1, CD49a, PDGFR-α/β, CD271	[81,82,83]
ADSCs	CD29, CD49e, CD44, CD144, CD13, CD73, CD90, CD105, CD146, CD10, CD36, CD106	[84,85]
DPSCs	CD13, CD29, CD44, CD59, CD73, CD90, CD105, CD146, OCT4, STRO-1, CD151, CD166	[80,86,87]
SHED	CD44, CD90, CD105, CD73, CD146, OCT4, STRO-1	[80,88]
PDLSCs	CD13, CD29, CD44, CD59, CD90, CD105, STRO-1	[80,89]
SCAP	CD13, CD44, CD24, CD29, CD73, CD90, CD105, CD106, CD146, STRO-1, OCT4, CD166	[80,90]
GMSCs	CD13, CD29, CD44, CD54, CD73, CD90, CD105, CD166, STRO-1	[91,92,93]
DFSCs	CD13, CD29, CD44, CD59, CD73, CD90, CD105, STRO-1	[80,94]

BM-MSCs, bone marrow mesenchymal stem cells; ADSCs, adipose-derived stem cells; DPSCs, dental pulp stem cells; SCAP, stem cells from apical papilla; PDLSCs, periodontal ligament stem cells; SHED, stem cells from *human* exfoliated deciduous teeth; GMSCs, gingival mesenchymal stem cells; DFSCs, dental follicle stem cells.

**Table 2:** Clinical trials of MSC-based therapies for cleft lip and palate registered in the ClinicalTrials.gov database.

Title	Trial Number	MSCs Source	Phase	Enrollment	Primary Purpose	Scaffold	Study Type
Use of Mesenchymal Stem Cells for Alveolar Bone Tissue Engineering for Cleft Lip and Palate Patients	NCT01932164	SHED	Phase 1, pilot	5	Treatment	Geistlich Bio-Oss^®♦^, III ^▲^	Interventional
Bone Tissue Engineering with Dental Pulp Stem Cells for Alveolar Cleft Repair	NCT03766217	SHED	Phase 3, pivotal	62	Treatment	Hydroxyapatite/collagen, III	Interventional
Tissue Engineered Constructs for Alveolar Cleft Repair	NCT03563495	BM-MSCs	Phase 1, pilot	10	Treatment	None	Interventional
The Effect of Bone Marrow Stem Cells Harvested from the Iliac Crest Versus Mandibular Ramus in Alveolar Cleft Regeneration	NCT06636643	BM-MSCs	Phase 1, pilot	12	N/A	Collagen sponge and nanohydroxyapatite, III	Observational
Validation of a Production Method of Stem Cell Isolated from the Nasal Cavity for an Innovative Cell Therapy of Cleft Palate	NCT02900014	Nasal MSCs	N/A *	2	Basic Science	None	Interventional
Cell Therapy for Craniofacial Bone Defects	NCT01616953	BM-MSCs	Phase 1 Phase 2, pilot	18	Treatment	None	Interventional

* N/A, not applicable. ^♦^ Geistlich Bio-Oss^®^ is manufactured by Geistlich Pharma AG, Wolhusen, Switzerland. ^▲^ III, Class III medical device. SHED, stem cells from *human* exfoliated deciduous teeth; BM-MSCs, bone marrow mesenchymal stem cells.

## Data Availability

No new data were created or analyzed in this study.

## Abbreviations

The following abbreviations are used in this review:

MSCs	mesenchymal stem cells
ASCs	adult stem cells
BM-MSCs	bone marrow mesenchymal stem cells
ESCs	embryonic stem cells
iPSCs	induced pluripotent stem cells
SuSCs	suture stem cells
ADSCs	adipose-derived stem cells
DPSCs	dental pulp stem cells
PDLSCs	periodontal ligament stem cells
DFSCs	dental follicle stem cells
SHED	stem cells from human exfoliated deciduous teeth
SCAP	stem cells from apical papilla
GMSCs	gingival mesenchymal stem cells
DSCs	dental stem cells
MACS	magnetic activated cell sorting
MHC	major histocompatibility complex
HLA	human leukocyte antigen
CL/P	cleft lip with or without cleft palate
SHED-CM	SHED conditioned medium
BMP2	bone morphogenetic protein 2
EVs	extracellular vesicles
PDL	periodontal ligament
TDM	treated dentin matrix
SD	Sprague-Dawley
HA/TCP	hydroxyapatite tricalcium phosphate
mDCs	monocyte-derived dendritic cells
PGE2	prostaglandin E2
IDO	indoleamine-pyrrole 2,3-dioxygenase
IFN-γ	interferon-γ
TNF-α	tumor necrosis factor-α
TGF-β	transforming growth factor-β
IL	interleukin
CCL-2	C-C motif chemokine ligand 2
Arg1	Arginase 1
VEGF	vascular endothelial growth factor
HGF	hepatocyte growth factor
CRISPR	clustered regularly interspaced short palindromic repeats
